# A Novel Multilevel-SVD Method to Improve Multistep Ahead Forecasting in Traffic Accidents Domain

**DOI:** 10.1155/2017/7951395

**Published:** 2017-02-05

**Authors:** Lida Barba, Nibaldo Rodríguez

**Affiliations:** ^1^Escuela de Ingeniería Informática, Pontificia Universidad Católica de Valparaíso, 2362807 Valparaíso, Chile; ^2^Facultad de Ingeniería, Universidad Nacional de Chimborazo, 060102 Riobamba, Ecuador

## Abstract

Here is proposed a novel method for decomposing a nonstationary time series in components of low and high frequency. The method is based on Multilevel Singular Value Decomposition (MSVD) of a Hankel matrix. The decomposition is used to improve the forecasting accuracy of Multiple Input Multiple Output (MIMO) linear and nonlinear models. Three time series coming from traffic accidents domain are used. They represent the number of persons with injuries in traffic accidents of Santiago, Chile. The data were continuously collected by the Chilean Police and were weekly sampled from 2000:1 to 2014:12. The performance of MSVD is compared with the decomposition in components of low and high frequency of a commonly accepted method based on Stationary Wavelet Transform (SWT). SWT in conjunction with the Autoregressive model (SWT + MIMO-AR) and SWT in conjunction with an Autoregressive Neural Network (SWT + MIMO-ANN) were evaluated. The empirical results have shown that the best accuracy was achieved by the forecasting model based on the proposed decomposition method MSVD, in comparison with the forecasting models based on SWT.

## 1. Introduction

Time series forecasting has reached high significance in planning and management for government institutions, industries, and business. Unfortunately the forecasting implementations are limited due to the data complexity. Environmental conditions, economy variables, risk situations, among others, are originated in highly dynamic systems; consequently their analysis becomes complex and inaccurate results have been often obtained.

From the literature review, several linear and nonlinear models have been proposed. One popular linear model is Autoregressive Integrated Moving Average (ARIMA), which was introduced by Box et al. [1] and widely applied to nonstationary time series such as, electricity consumption [2], rainfall [3], solar radiation [4], and tourists arrivals [5]. Empirical results have shown varied accuracy levels by testing different parameter configuration; therefore the performance of ARIMA is dependent on an effective selection of parameters. Even more ARIMA models are limited to deal with constant variance processes and normally distributed residuals which are rarely satisfied in real life signals.

On the other hand, the Artificial Neural Networks (ANNs) are nonparametric models that have been implemented for modeling nonstationary time series. The nonlinear features of the ANNs sometimes explain the nonlinear relationships among the explaining variables and observed phenomena. By instance, Li and Shi (2010) applied three typical ANN techniques for one-step ahead wind speed forecasting by using different datasets of two representative north American sites [6], by implementation of Feed Forward Back-Propagation (FFBP), Radial Basis Function (RBF), and Adaptive Linear Element (ADALINE). After multiple tests the FFBP model was considered the best model for one site, while the RBF model was the best for other sites; therefore the research concludes that it is not recommended to employ only one type of ANN model in wind speed forecasting. Other representative example is the prices variation range; Laboissiere et al. [7] modeled stock prices of power distribution companies through an ANN based on Levenberg-Marquardt (LM). Different Multilayer Perceptron (MLP) topologies were evaluated iteratively with opening and closing prices and other correlated variables as input, different number of hidden neurons and one output until finding the best configuration for short-term horizon. In general, an ANN implementation implies taking some decisions after several tests, such as, network topology, signal propagation method, activation function, weights updating, hidden levels, and numbers of nodes. Sometimes higher accuracy can be reached by an ANN, but this leads a computational complexity increasing [8, 9].

A novel solution is the hybrid models which are based on the combination of techniques. Preprocessing methods combined with conventional linear and nonlinear models are implemented to improve the forecast. The Wavelet Decomposition (WD) was originated in 1984 with the discovery of Grossman and Morlet in the quantum physics context [10]. The conjunction Wavelet Decomposition and artificial intelligence can improve the efficiency of pure models in many areas such as, hydrology [11, 12], transportation systems [13], and public health [14].

In this work is proposed a new decomposition method based on Multilevel Singular Value Decomposition (MSVD) for extraction components of low and high frequency of a nonstationary time series in order to improve the accuracy of a linear and a nonlinear forecasting model. A Multiple Input and Multiple Output Autoregressive (MIMO-AR) model is implemented based on MSVD. Three relevant traffic accidents' time series of Santiago, Chile, are used to evaluate the forecast performance. The MSVD + MIMO-AR joint model is validated through comparisons with respect to the performance of Stationary Wavelet Decomposition combined with MIMO-AR (SWT + MIMO-AR) and SWT combined with an Autoregressive Neural Network based on Levenberg-Marquardt (SWT + MIMO-ANN).

Related works about traffic accidents forecasting are scarce; most applications make classification with multivariate methods [15–18]. There have been found some forecast applications related to transportation areas, such as, traveling time [19], traffic flow parameters as volume, travel speeds and occupancies [20, 21], the market demand after transportation disruptions [22], and freight transportation demand [23, 24].

This paper is organized as follows.  Section 2 describes the proposed methodology based on MSVD + MIMO-AR.  Section 3 describes SWT + MIMO-AR and SWT + MIMO-ANN.  Section 4 presents the efficiency metrics.  Section 5 specifies the study case.  Section 6 shows the empirical research results. Finally Section 7 concludes the paper.

## 2. Forecasting Methodology Based on MSVD and MIMO-AR

The proposed forecasting methodology is described in two stages; the first stage is presented by Multilevel Singular Value Decomposition to decompose a time series into two components of low and high frequency, whereas the second stage performs the prediction through the MIMO-AR model. The MIMO-AR inputs are the lagged values of the components extracted, and the outputs are the prediction for multiple horizon.

### 2.1. Multilevel Singular Value Decomposition

MSVD is a method inspired in the pyramidal process implemented in multiresolution analysis of Mallat Algorithm [25] which was defined for wavelet representation. In this method is proposed the multilevel decomposition of a Hankel matrix, at difference of standard HSVD [26]. MSVD implements iterative embedding and pyramidal decomposition with a fixed window length *L* = 2; therefore two components are obtained at each decomposition level.

MSVD algorithm is summarized as the pseudocode shown in Algorithm 1. The input algorithm is the observed time series *x* of length *N*, and at the end, two additive and intrinsic components are obtained as outputs, *c*_*L*_ and *c*_*H*_, which represent the Low Frequency and the High Frequency Component, respectively, each one of length *N*. MSVD is performed in three steps:* embedding* through a Hankel matrix of 2 × (*N* − 1) dimension as (2),* decomposition *in orthogonal matrices of eigenvectors *U* and *V* and singular values *λ*_1_, *λ*_2_, and finally* extraction *from elementary matrices *H*_1_ and *H*_2_.

MSVD is processed iteratively until the optimum decomposition level *J*, when the Singular Spectrum Rate Δ*R* reaches the asymptotic point. Equations (1a) and (1b) describe the computation of Δ*R*, which is based on the relative energy of the singular values *R*_*j*_ (for *j* = 1,…, *k* decomposition levels).

(1a) ΔRj=RjRj+1,

(1b) Rj=λ1λ1+λ2,

(2) H=x1x2⋯xN−1x2x2+1⋯xN.

### 2.2. Multiple Input Multiple Output Autoregressive Prediction

The AR model is implemented to forecast the time series by using the MIMO strategy. MIMO is used for overcoming the error accumulation problem that is observed in the recursive strategy and the direct strategy and for preserving the random relationships between predicted values [27]. MIMO-AR computes the output for the forecast horizon in a single simulation with unique model, which returns a vector rather than a scalar value as follows:

(3) x^n+1,x^n+2,…,x^n+τ=fzn,zn−1,…,zn−P+1,

where *τ* is the forecast horizon and $\hat{x}$ is the matrix of outputs. Each column will contain the prediction related to a specific horizon.

The following equation defines MIMO in matrix form:

(4) $$\begin{array}{c} \hat{x}=\beta z^{T}, \end{array}$$

where *β* is a *τ* × 2*P* matrix of linear coefficients and *z*^*T*^ is the Autoregressive transposed matrix created from the components *c*_*L*_ and *c*_*H*_ that were extracted by means of MSVD. Matrix *z* is *N*_*t*_ × 2*P* dimension, where *N*_*t*_ is the number of samples. The matrix of coefficients *β* is computed with the Least Square Method (LSM) as below:

(5) $$\begin{array}{c} \beta =x\times z^{†}, \end{array}$$

where *z*^†^ is the Moore-Penrose pseudoinverse matrix of *z*.

## 3. Stationary Wavelet Transform Combined with MIMO-AR and Stationary Wavelet Transform Combined with MIMO-ANN

### 3.1. Stationary Wavelet Transform

Stationary Wavelet Transform (SWT) is improved version of Discrete Wavelet Transform. SWT is also known in the literature as Dyadic Wavelet Transform, Maximal Overlap Transform, Undecimated Discrete Wavelet Transform, and Redundant Wavelet Transform. The implementation of SWT is defined in the algorithm of Shensa [28]. SWT implements filtering but the downsampling procedure is omitted and the filters are upsampled [29, 30].

In SWT the length of the observed signal must be an integer multiple of 2^*j*^, where *j* = 1,2,…, *J* is the scale number. The signal is separated in approximation coefficients and detail coefficients at different scales; this hierarchical process is called multiresolution decomposition [25].

**Figure 1:**
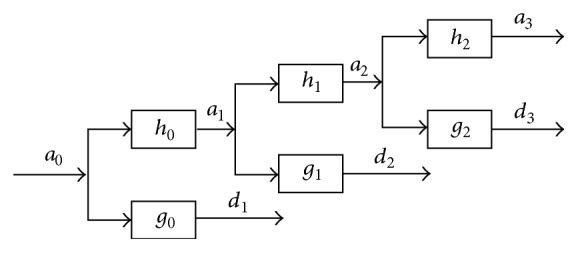
Decomposition scheme of SWT with *J* = 3.

The observed signal *a*_0_ (which was named *x* in previous section) is decomposed in approximation and detail coefficients through a bank of low pass filters (*h*_0_, *h*_1_,…, *h*_*J*−1_) and a bank of high pass filter (*g*_0_, *g*_1_,…, *g*_*J*−1_) one to each level as the scheme of Figure 1. Each level filter is upsampled version of the previous one. The components obtained after the decomposition are never decimated; therefore they have the same length as the observed signal.

At first decomposition level the observed signal *a*_0_ is convoluted with the first low pass filter *h*_0_ to obtain the first approximation coefficients *a*_1_ and with the first high pass filter *g*_0_ to obtain the first detail coefficients *d*_1_. The process is defined as follows:

(6a) a1n=∑ih0ia0n−i,

(6b) d1n=∑ig0ia0n−i.

The process follows iteratively, for *j* = 1,…, *J* − 1 and it is given as

(7a) aj+1n=∑ihjiajn−i,

(7b) dj+1n=∑igjiajn−i.

Inverse Stationary Wavelet Transform (iSWT) performs the reconstruction. The implementation of iSWT consists in applying the operations that were performed in SWT but in inverse order and based on equivalent filters of reconstruction. SWT obtains subbands of frequency; the last approximation coefficient reconstructed ${\tilde{a}}_{J}$ gives rise to the component of low frequency *c*_*L*_, whereas all reconstructed detail coefficients ${\tilde{d}}_{j}$ are added to obtain the component of high frequency *c*_*H*_.

### 3.2. Forecast Based on Components Extracted by SWT

The forecasting is implemented via both a linear and nonlinear models to evaluate the performance of the decomposition obtained through SWT.

The MIMO-AR model has the same structure that was used in the previous section. Therefore the nonlinear forecast based on an Artificial Neural Network is described here.

A sigmoid Multilayer Perceptron (MLP) of three layers [31] is implemented. The ANN is denoted with *ANN*(*P*, *Q*, *h*). The inputs are the *P* lagged terms contained in the regressor matrix *z*. The hidden layer has *Q* nodes and the output has *h* nodes, which are the forecast horizon. The prediction at each forecast horizon ${\hat{x}}_{n\;+\;h}$ via the ANN is expressed with,

(8a) x^n+h=∑j=1QbjhYj,

(8b) Yj=f∑i=1Pwijzi,

where *h* = 1,…, *τ*, *b*_*jh*_ is the weight of the connection between *j*th hidden node and *h*th output, and *Y*_*j*_ is *j*th hidden level output, while *w*_*ij*_ is the weight of the connection between *i*th input node and *j*th hidden node, and *z*_*i*_ represents *i*th lagged vector.

The sigmoid transfer function is denoted with the following:

(9) $$\begin{array}{c} f\left(\;x\;\right)=\frac{1}{1+e^{-x}}. \end{array}$$

Parameters *b* and *w* are updated with the application of the learning algorithm, in this case, Levenberg-Marquardt [32, 33].

## 4. Efficiency Metrics

The performance of the models MSVD + MIMO-AR, SWT + MIMO-AR, and SWT + MIMO-ANN is evaluated with three efficiency criteria. The normalized Root Mean Square Error (nRMSE), the modified Nash-Suctliffe Efficiency (mNSE) [34], and the modified Index of Agreement (mIA) [35]. Metrics mNSE and mIA at difference of nRMSE are not based on square differences; in place they are based on the Sum of Absolute Error (SAE) and the Sum of Absolute Deviation (SAD); the formulas are shown below:

(10) nRMSE=1/Nt∑i=1Ntxi−x^i2x¯

(11a) SAE=∑i=1Ntx^i−xi,

(11b) SAD=∑i=1Ntxi−x¯,

where *x*_*i*_ is *i*th observed value, ${\hat{x}}_{i}$ is *i*th predicted value, $\bar{x}$ is the mean of all *x*_*i*_, and *N*_*t*_ is the testing sample size.

Scores mNSE and mIA overcome the possible oversensitivity to extreme values induced by metrics based on square computation and increase the possible sensitivity for lower values.

(12) mNSE=1−SAESAD,

(13a) mIA=1−SAE2SAD,if  SAE≤2SAD,

(13b) mIA=2SADSAE−1,if  SAE>2SAD.

Metrics mNSE and mIA are monotonically and functionally related, but the use of 2SAD balances the number of deviations evaluated within the numerator and within the denominator of the factional part.

## 5. Case Study

The Chilean Police and the National Traffic Safety Commission (CONASET) are the official institutions that register the data of traffic accidents in Chile [36]. The data are continuously collected, and in this study they have been sampled with a fixed interval of 7 days (one week). Three discrete time series of injured people in traffic accidents in Santiago from 2000:1 to 2014:12 due to different causes are used. CONASET has defined one hundred causes of traffic accidents; in this study case the series of persons with injuries I-G1 and I-G2 group include 20 causes which are related to improper behavior of drivers, passengers, and pedestrians, with an incidence rate of 75%, whereas the series I-G3 groups include the remaining causes (not related to improper behavior) with an incidence rate of 25%.  Table 1 presents the series of persons with injuries in traffic accidents and the causes related to the incidents.

**Figure 2:**
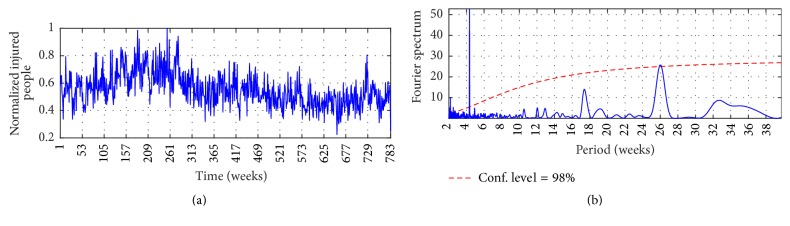
(a) I-G1 and (b) FPS of I-G1. The thick solid line is the global wavelet spectrum for I-G1, while the dashed line is the red-noise spectrum.

**Figure 3:**
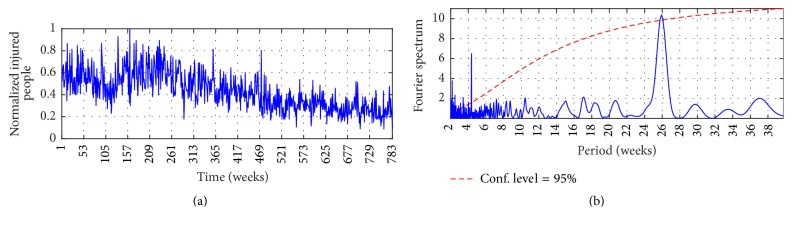
(a) I-G2 and (b) FPS of I-G2. The thick solid line is the global wavelet spectrum for I-G1, while the dashed line is the red-noise spectrum.

**Figure 4:**
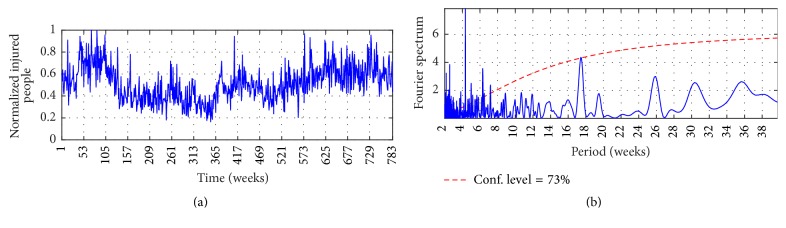
(a) I-G3 and (b) FPS of I-G3. The thick solid line is the global wavelet spectrum for I-G1, while the dashed line is the red-noise spectrum.

Figures 2(a), 3(a), and 4(a) show the observed time series, whereas Figures 2(b), 3(b), and 4(b) show the Fourier Power Spectrum (FPS) of I-G1, I-G2, and I-G3, respectively. High data variability is observed during the analyzed period; by instance I-G1 presents upward trend between weeks 1 and 280 which is followed by downward trend until week 348; that behavior continues in the remaining observed period. On the other hand, I-G2 presents downward trend from week 232 until the end, and I-G3 presents upward trend from week 491 until end. The FPS analysis shows the signal spectrum and the red-noise spectrum; a signal spectrum peak is significative when its value is higher than the red-noise spectrum [37]. Both series, I-G1 and I-G2, present the highest peak at week 26 at 98% of confidence level, whereas I-G3 shows the highest peak at week 17 at 73% of confidence level. The order of each AR model was selected with the number of weeks for which the highest power spectrum was found; consequently *P* = 26 for I-G1 and I-G2, and *P* = 17 for I-G3.

## 6. Empirical Research Result

The empirical results obtained by the application of MSVD + MIMO-AR, SWT + MIMO-AR, and SWT + MIMO-ANN are presented in this section in two stages decomposition and prediction.

### 6.1. Decomposition Based on MSVD and SWT

**Figure 5:**
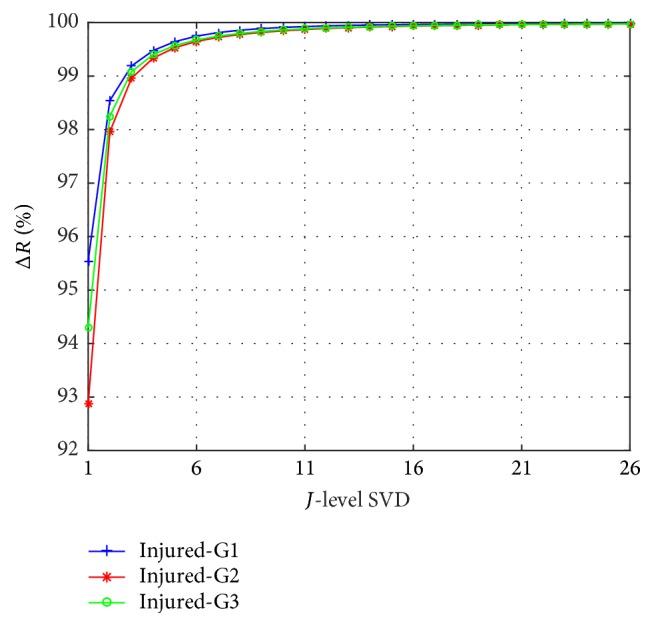
Decomposition levels versus Singular Spectrum Rate.

MSVD implements an iterative process which finishes when Δ*R* reaches the asymptotic value. The Singular Spectrum Rate Δ*R* for each decomposition level *J* is illustrated in Figure 5; the asymptotic value is reached when Δ*R* ≈ 1, and it was observed in repetition 16. Therefore the iterative process finishes when iteration 16 was performed; this condition is used with all-time series.

SWT decomposition is implemented trough Daubechies of order 2 (Db2) (due to the inaccurate results that were obtained with the other types of wavelet functions, they are not presented). Three decomposition levels (*J* = 3) were selected according to the period fluctuation between 8 and 16 weeks.

**Figure 6:**
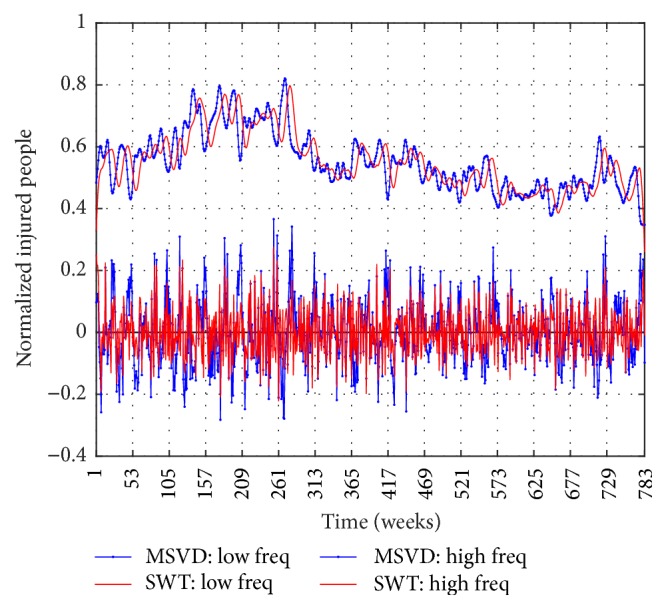
I-G1, Low Frequency Component and High Frequency Component via MSVD and SWT.

**Figure 7:**
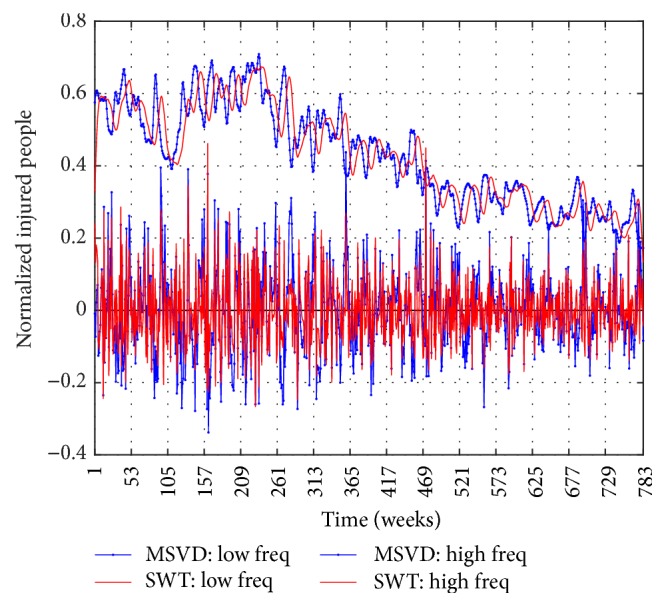
I-G2, Low Frequency Component and High Frequency Component via MSVD and SWT.

**Figure 8:**
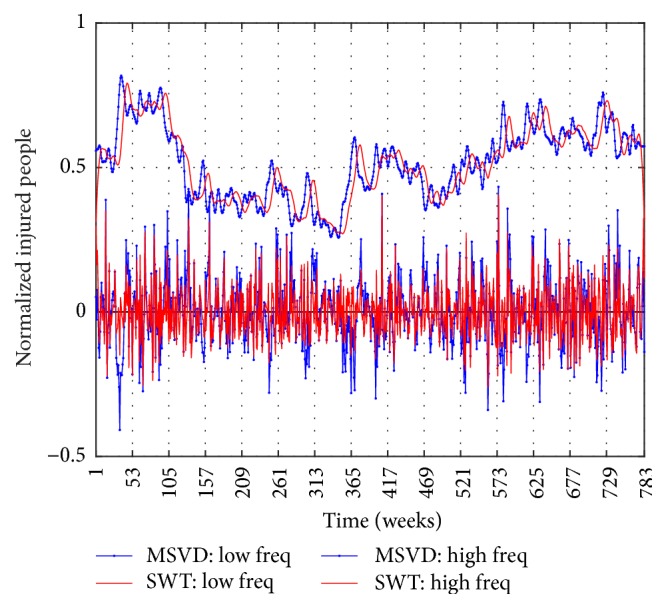
I-G3, Low Frequency Component and High Frequency Component via MSVD and SWT.

Figures 6, 7, and 8 show the components of low frequency and high frequency obtained with MSVD and SWT for I-G1, I-G2, and I-G3, respectively. The *c*_*L*_ components extracted by both MSVD and SWT show long-memory periodicity features, whereas the *c*_*H*_ components show short-term periodic fluctuations.

### 6.2. Prediction via MIMO-AR and MIMO-ANN Models

The MIMO strategy is implemented to predict the number of injured people in traffic accidents for multiple horizon through the Autoregressive model and through an Artificial Neural Network. For both linear and nonlinear models the spectral analysis developed by means of FPS informs about the order of the models; it was shown in Figures 2(b), 3(b), and 4(b). The inputs are the *P* lagged values of *c*_*L*_ and the *P* lagged values of *c*_*H*_, and the outputs are the number of injured people for the next *τ* weeks. The components *c*_*L*_ and *c*_*H*_ were extracted previously via MSVD and SWT.

Before prediction, each data set of low and high frequency has been divided into two subsets, training and testing. The training subset (*N*_tr_) involves 70% of the samples, and consequently the testing subset (*N*_*t*_) involves the remaining 30%.

The MIMO-ANN structure denoted with (*P*, *Q*, *h*) was implemented with *P* lagged values (set previously with the FPS information), *Q* = log_2_(*N*_tr_), where *N*_tr_ is the training subset size, and *h* = 8 forecast horizon.

The prediction performance is evaluated with the efficiency metrics nRMSE, mNSE, and mIA, which are presented in Tables 2, 3, and 4 for I-G1, I-G2, and I-G3, respectively. The ANN results correspond to 500 epochs and 10 runs. The results obtained through the nonlinear model SWT + MIMO-ANN are inferior with respect to the linear models MSVD + MIMO-AR and SWT + MIMO-AR; therefore the rest of comparisons are performed between the linear models. The SWT + MIMO-AR results for 14 weeks' ahead prediction of all-time series are not presented due to the poor results that were obtained, as those results that were obtained with SWT + MIMO-ANN for forecast horizon higher than 8 weeks.

**Figure 9:**
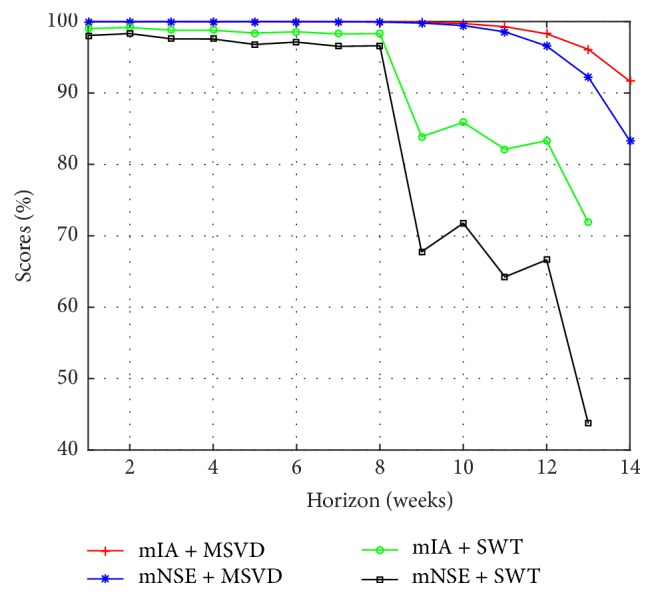
Multistep forecasting results, comparison for I-G1.

From Table 2 and Figure 9, MSVD + MIMO-AR obtains the best accuracy in the forecast of I-G1. A significative gain was observed in each forecasting horizon of the model based on MSVD with respect to the models based on SWT. The mean gain for 1 to 13 weeks of MSVD + MIMO-AR over SWT + MIMO-AR is 17.7% in mNSE and 8.1% in mIA.

**Figure 10:**
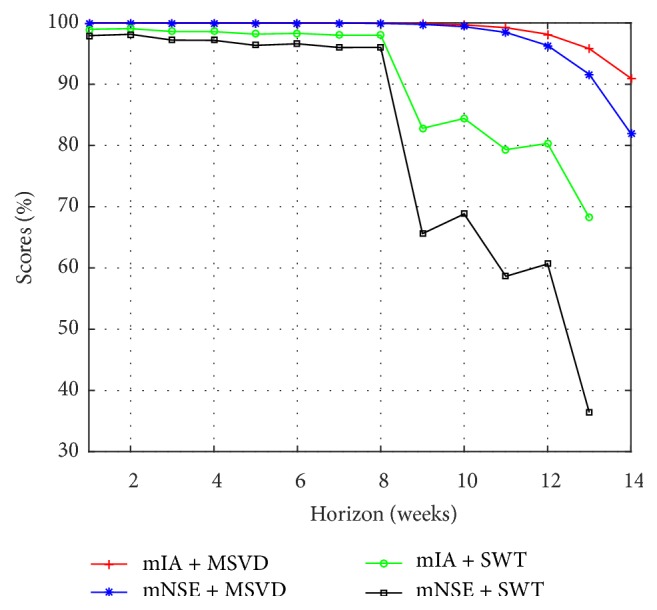
Multistep forecasting results, comparison for I-G2.

From Table 3 and Figure 10, MSVD + MIMO-AR obtains the best accuracy in the forecasting of I-G2. A significative gain of MSVD + MIMO-AR was observed in each forecasting horizon with respect to the models based on SWT. The mean gain for 1 to 13 weeks of MSVD + MIMO-AR over SWT + MIMO-AR is 20.6% in mNSE and 9.3% in mIA.

**Figure 11:**
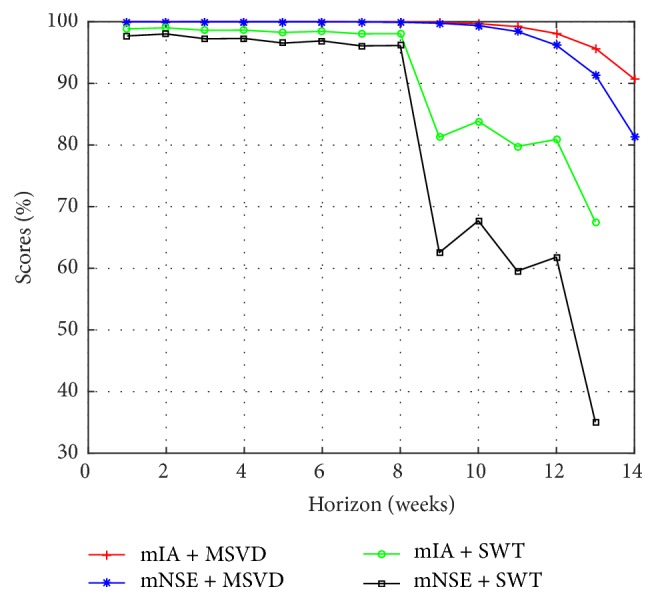
Multistep forecasting results, comparison for I-G3.

From previous analysis that was made for I-G1 and I-G2, from Table 4 and Figure 11, the I-G3 forecast based on MSVD + MIMO-AR is also more accurate than the prediction obtained with the models based on SWT. The mean gain for 1 to 13 weeks of MSVD + MIMO-AR over SWT + MIMO-AR is 20.9% in mNSE and 9.4% in mIA.

**Figure 12:**
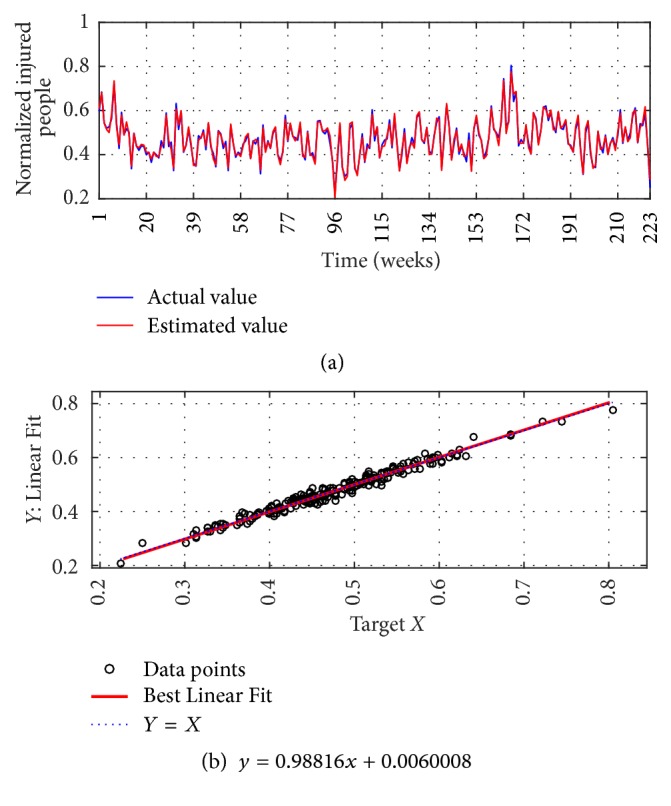
I-G1 Prediction by MSVD + MIMO-AR. (a) Observed versus predicted and (b) Linear Fit.

**Figure 13:**
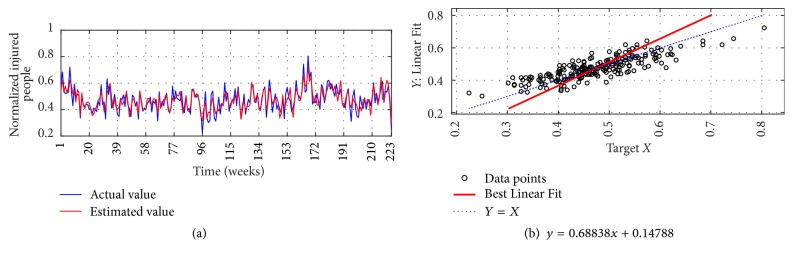
I-G1 Prediction by SWT + MIMO-AR. (a) Observed versus predicted and (b) Linear Fit.

The I-G1 Prediction via MSVD + MIMO-AR for 14 weeks' ahead prediction is shown in Figures 12(a) and 12(b); from figures good fit is observed between actual and estimated values. Metrics computation gives nRMSE of 2.9%, mNSE of 83.3%, and mIA of 91.6%. The prediction of the same series via SWT + MIMO-AR for 13 weeks' ahead prediction is shown in Figure 13; lower accuracy is observed with nRMSE of 10.1%, mNSE of 43.7%, and mIA of 71.8%.

**Figure 14:**
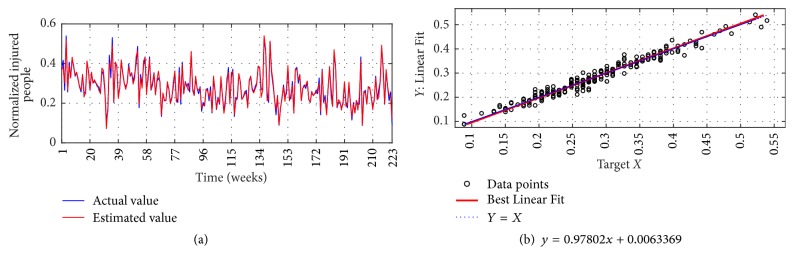
I-G2 Prediction by MSVD + MIMO-AR. (a) Observed versus predicted and (b) Linear Fit.

**Figure 15:**
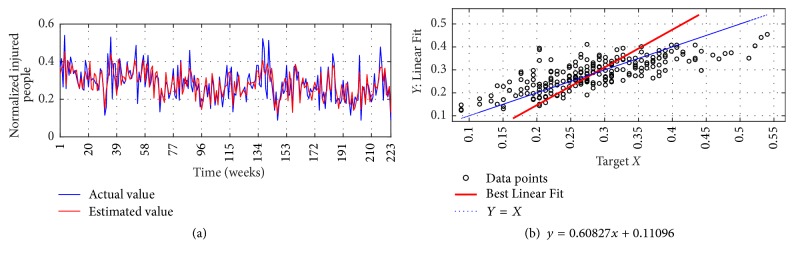
I-G2 Prediction by SWT + MIMO-AR. (a) Observed versus predicted and (b) Linear Fit.

The I-G2 Prediction via MSVD + MIMO-AR for 14 weeks' ahead prediction is shown in Figures 14(a) and 14(b); from figures good fit is observed with nRMSE of 5.8%, mNSE of 81.9%, and mIA of 90.9%. The prediction of the same series via SWT + MIMO-AR for 13 weeks' ahead prediction is shown in Figure 15, lower accuracy is observed with nRMSE of 19.6%, mNSE of 36.4%, and mIA of 68.2%.

**Figure 16:**
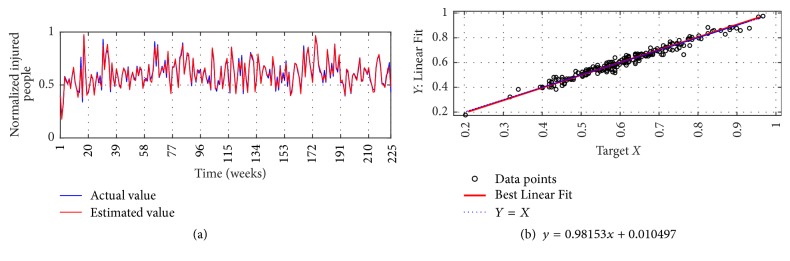
I-G3 Prediction by MSVD + MIMO-AR. (a) Observed versus predicted and (b) Linear Regression, fitted line.

**Figure 17:**
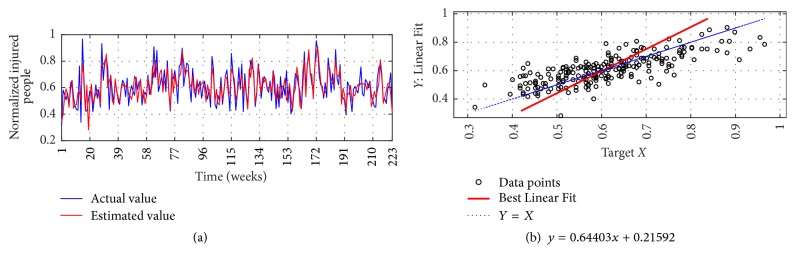
I-G3 Prediction by SWT + MIMO-AR. (a) Observed versus predicted and (b) Linear Regression, fitted line.

The I-G3 Prediction via MSVD + MIMO-AR for 14 weeks' ahead prediction is shown in Figures 16(a) and 16(b); from figures good fit is observed with nRMSE of 3.8%, mNSE of 81.4%, and mIA of 90.7%. The prediction of the same series via SWT + MIMO-AR for 13 weeks' ahead prediction is shown in Figure 17, lower accuracy is observed with nRMSE of 13.0%, mNSE of 35.0%, and mIA of 67.5%.

## 7. Conclusions

In this paper was presented a new decomposition method for extracting components of low and high frequency of a nonstationary time series. The method was called MSVD due to the use of Multilevel Singular Value Decomposition of a Hankel matrix. MSVD was evaluated for multistep ahead forecasting based on the Autoregressive model and the MIMO strategy.

The forecasting model MSVD + MIMO-AR was compared with a linear and a nonlinear forecast model based on the commonly decomposition technique Stationary Wavelet Transform. The empirical application was developed through three time series coming from traffic accidents domain. All experiments have shown the superior accuracy of MSVD + MIMO-AR with respect to SWT + MIMO-AR and SWT + MIMO-ANN. MSVD + MIMO-AR in comparison with the second best model SWT + MIMO-AR, achieving mean mNSE-gain of 19.8% and mean mIA-gain of 8.9% for 13 weeks' ahead forecasting of persons with injuries in traffic accidents in Santiago, Chile. It was also observed that an ANN with Levenberg-Marquardt based on SWT decays significantly from 9 weeks' ahead forecasting.

Furthermore, the implementation of MSVD presents simplicity with respect to other techniques based on singular values by the use of a fixed window length in the embedding step, and although the algorithm is iterative, the stopping condition is guaranteed by the convergence of the Singular Spectrum Rate parameter. MSVD also presents more simplicity with respect to SWT; this is because SWT requires taking some decisions to select the wavelet mother function.

Future implementations will consider new application areas to support planning and management tasks of public and private institutions.

## Figures and Tables

**Algorithm 1:**
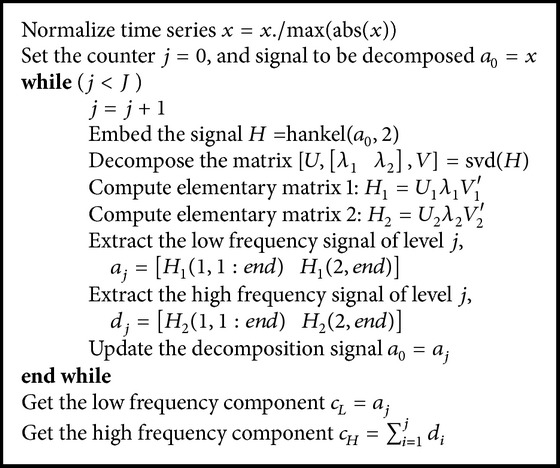
Multilevel SVD algorithm.

**Table 1:** Series of injured people in traffic accidents due to different causes.

Series	Causes	Incidence rate
I-G1	Unwise distance, inattention to traffic conditions, disrespect to red light,	60%
	disrespect to pedestrian passing, disrespect to stop sign,	
	disrespect to giving way sign, pedestrian crossing the road suddenly,	
	drunk driver, disrespect for giving the right of way,	
	and unexpected change of track	

I-G2	Overtaking without enough time or space, improper turns,	15%
	driving under the influence of alcohol, backward driving,	
	driving in opposite direction, recklessness in pedestrian,	
	pedestrian outside the allowed crossing, improper speed,	
	alcohol in pedestrian, and passenger gets in or gets out of a moving vehicle	

I-G3	Road deficiencies, mechanical failures, undetermined causes, and noncategorized causes	25%

**Table 2:** Forecasting results for I-G1.

h (week)	nRMSE			mNSE(%)			mIA(%)		
	MSVD + AR	SWT + AR	SWT + ANN	MSVD + AR	SWT + AR	SWT + ANN	MSVD + AR	SWT + AR	SWT + ANN
1	0.0021	0.34	0.22	99.9	98.0	98.8	99.9	99.0	99.4
2	0.0030	0.29	0.19	99.9	98.3	98.9	99.9	99.2	99.5
3	0.0007	0.44	0.29	99.9	97.6	98.4	99.9	98.8	99.2
4	0.0030	0.44	0.60	99.9	97.6	96.7	99.9	98.8	98.3
5	0.0003	0.56	1.49	99.9	96.8	91.7	99.9	98.4	95.8
6	0.0024	0.51	3.10	99.9	97.1	82.9	99.9	98.6	91.4
7	0.0041	0.63	5.19	99.9	96.5	71.7	99.9	98.3	85.8
8	0.0125	0.61	7.07	99.9	96.6	63.3	99.9	98.3	81.6
9	0.0366	5.6	—	99.8	67.8	—	99.9	83.9	—
10	0.0990	4.8	—	99.4	71.6	—	99.7	85.9	—
11	0.2512	6.5	—	98.5	64.2	—	99.3	82.1	—
12	0.6009	5.9	—	96.6	66.6	—	98.3	83.3	—
13	1.3685	10.13	—	92.2	43.7	—	96.1	71.8	—
14	2.9872	—	—	83.3	—	—	91.6	—	—

Min	0.0003	0.29	0.19	83.3	43.7	63.3	91.6	71.8	81.6
Max	2.9872	10.1	7.07	99.9	98.3	98.9	99.9	99.2	99.5
Mean 1–8	0.003	0.48	2.3	99.9	97.3	87.7	99.9	98.7	93.9
Mean 1–13	0.1834	2.8	—	98.9	84.1	—	98.9	92.0	—
Mean 1–14	0.3837	—	—	97.8	—	—	98.9	—	—

**Table 3:** Forecasting results for I-G2.

h (week)	nRMSE			mNSE(%)			mIA(%)		
	MSVD + AR	SWT + AR	SWT + ANN	MSVD + AR	SWT + AR	SWT + ANN	MSVD + AR	SWT + AR	SWT + ANN
1	0.00048	0.62	0.91	99.9	97.9	97.1	99.9	98.9	98.5
2	0.00009	0.56	0.84	99.9	98.1	97.2	99.9	99.1	98.6
3	0.00132	0.85	1.64	99.9	97.2	94.4	99.9	98.6	97.2
4	0.00058	0.83	3.84	99.9	97.2	87.1	99.9	98.6	93.5
5	0.00105	1.12	8.68	99.9	96.3	71.4	99.9	98.2	85.7
6	0.00249	1.05	13.78	99.9	96.6	55.1	99.9	98.3	77.6
7	0.00761	1.22	14.67	99.9	95.9	47.2	99.9	97.9	73.6
8	0.02418	1.18	15.34	99.9	96.0	42.7	99.9	98.0	71.3
9	0.07071	10.4	—	99.8	65.6	—	99.9	82.8	—
10	0.19062	9.5	—	99.4	68.8	—	99.7	84.4	—
11	0.48291	12.5	—	98.5	58.6	—	99.2	79.3	—
12	1.15837	11.6	—	96.3	60.6	—	98.2	80.3	—
13	2.64161	19.6	—	91.6	36.4	—	95.8	68.2	—
14	5.77482	—	—	81.9	—	—	90.9	—	—

Min	0.00009	0.56	0.84	81.9	36.4	42.7	90.9	68.2	71.3
Max	5.77482	19.6	15.3	99.9	98.1	97.2	99.9	99.1	98.6
Mean 1–8	0.004	0.93	7.5	99.9	96.9	74.0	99.9	98.5	87.0
Mean 1–13	0.35	5.5	—	98.9	81.9	—	99.4	90.9	—
Mean 1–14	0.74	—	—	97.7	—	—	98.8	—	—

**Table 4:** Multistep MIMO forecasting results, nRMSE, mNSE, and mIA for I-G3.

h (week)	nRMSE			mNSE(%)			mIA(%)		
	MSVD + AR	SWT + AR	SWT + ANN	MSVD + AR	SWT + AR	SWT + ANN	MSVD + AR	SWT + AR	SWT + ANN
1	0.0001	0.48	0.46	99.9	97.7	97.6	99.9	98.9	98.8
2	0.0020	0.40	0.41	99.9	98.0	97.9	99.9	99.0	98.9
3	0.0005	0.57	0.52	99.9	97.2	97.3	99.9	98.6	98.6
4	0.0007	0.57	1.58	99.9	97.3	91.9	99.9	98.6	95.9
5	0.0004	0.72	3.76	99.9	96.5	81.4	99.9	98.3	90.7
6	0.0015	0.63	7.03	99.9	96.9	65.6	99.9	98.4	82.8
7	0.0052	0.82	9.54	99.9	96.1	53.2	99.9	98.0	76.6
8	0.0165	0.82	9.63	99.9	96.1	52.4	99.9	98.1	76.2
9	0.0482	7.83	—	99.8	62.5	—	99.9	81.3	—
10	0.1303	6.59	—	99.4	67.7	—	99.7	83.9	—
11	0.3293	8.44	—	98.4	59.6	—	99.2	79.8	—
12	0.7864	7.96	—	96.2	61.8	—	98.1	80.9	—
13	1.7868	13.04	—	91.4	35.0	—	95.7	67.5	—
14	3.8944	—	—	81.4	—	—	90.7	—	—

Min	0.0001	0.56	0.41	81.4	35.0	52.4	90.7	67.5	76.2
Max	3.89	19.6	9.6	99.9	98.0	97.9	99.9	99.0	98.9
Mean 1–8	0.003	0.63	4.12	99.9	96.9	79.7	99.9	98.5	89.8
Mean 1–13	0.24	5.46	—	98.9	81.7	—	99.4	90.9	—
Mean 1–14	0.5	—	—	97.6	—	—	98.8	—	—
